# Scaling Relationships between Leaf Mass and Total Plant Mass across Chinese Forests

**DOI:** 10.1371/journal.pone.0095938

**Published:** 2014-04-23

**Authors:** Shanshan Xu, Yan Li, Genxuan Wang

**Affiliations:** The state key laboratory of plant physiology and biochemistry, Institute of ecology, College of Life Sciences, Zhejiang University, Hangzhou, China; Tennessee State University, United States of America

## Abstract

Biomass partitioning is important for illustrating terrestrial ecosystem carbon flux. West, Brown and Enquist (WBE) model predicts that an optimal 3/4 allometric scaling of leaf mass and total biomass of individual plants will be applied in diverse communities. However, amount of scientific evidence suggests an involvement of some biological and environmental factors in interpreting the variation of scaling exponent observed in empirical studies. In this paper, biomass information of 1175 forested communities in China was collected and categorized into groups in terms of leaf form and function, as well as their locations to test whether the allocation pattern was conserved or variable with internal and/or environmental variations. Model Type II regression protocol was adopted to perform all the regressions. The results empirically showed that the slopes varied significantly across diverse forested biomes, between conifer and broadleaved forests, and between evergreen and deciduous forests. Based on the results, leaf form and function and their relations to environments play a significant role in the modification of the WBE model to explore more accurate laws in nature.

## Introduction

Metabolism, which dictates material and energy fluxes through organisms, is a fundamental process in plant life [1]. As the basis for many life-history strategies and ecological theories interested in ecological dynamics and global change, the scaling exponent for the partitioning of total body biomass among metabolic organs is of major interest in basic and applied ecology [2]–[6]. Foliar leaves are the principle or sole light-harvesting organs for metabolic reactions in photosynthesis, which is the primary metabolic activity involved in carbon fluxes. Therefore, a quantitative understanding of biomass patterns of leaf biomass to total mass is of fundamental importance to terrestrial carbon cycle. WBE model (West, Brown and Enquist, hence WBE) has outlined a set of allometric derivations interrelating individual plant growth as well as vegetative organ biomass partitioning, covering the broadest allometric phenomena at multiply scales of ecological organizations [1], [7]. Based on the constraints of maximizing photosynthetic harvesting capacity and resource transport while minimizing hydrodynamic resistance and transport times, the allometric partitioning model predicts that allometric relationships are governed by 1/4 power rules and leaf mass should scale to the 3/4^th^ power of total mass [1], [4], [7]–[12]. Extensive data for conifers, monocots and dicots spanning 12 orders of magnitude fit the predicted relationships remarkably well, with two empirical exponents of 0.739 (95% CIs: 0.646–0.831) for angiosperms and 0.756 (95% CIs: 0.664–0.846) for gymnosperms, both of which were very close to 3/4 [13], [14]. Although the 3/4 scaling exponent has been sustained in many theoretical and empirical studies, an extension of WBE model based on the “fractal continuum” theory predicts that the intraspecific allometry of plant biomass partitioning should fall along a continuum of variation rather than being a single optimal exponent [15]. Nevertheless, deviated from the predictions, a lot of variations in plant allometry have been observed in empirical studies [16]–[28] and discussed in reviews [29]–[31]. These variations are assumed to result from altering multiple and/or different physiological and morphological factors in response to ontogeny [17], [24], [27], [28], environmental variability [21], [25], [26] and species specificity [16], [22], [23], [30]. The universal applications of the WBE are challenged [33]–[35].

In contrast to the central assumption of WBE model that leaf properties keep consistent with whole-plant size across orders of magnitude of plant size [7], [10], [36], leaves do vary in form and function with species variety and environmental heterogeneity. As both cause and consequence for plant adaptive strategy, leaves always have different chlorophyll concentrations, light compensation points and light use efficiencies in response to environmental variation or phyletic affiliation [20]. These variations in leaf properties are commonly present in nature and influence plant behavior at a variety of levels of complexity. In addition, leaves are more variable when compared with other component biomass, ascribed to the high sensitivity of leaf mass to light, water, nutrient and soil conditions [37], [38]. Therefore, variability in allometry of plant biomass partitioning can be understood by considering specific leaf growth forms or functional groups. Leaf habit and leaf form are vital leaf properties, the substantial difference of which could exert profound impact on populations and communities with interactions of heterogeneous environmental availability. For example, plants in harsh environments prefer relatively high ratio of photosynthetic mass to body mass as a consequence of thicker leaves and relatively smaller body size [21]; in tropical dry forests, broadleaved deciduous trees tend to have greater nutrient resorption efficiencies, specific leaf area and photosynthetic rate compared with broadleaved evergreen trees [39]; boreal coniferous forests are prone to show lower scaling law of *M*_L_ with *M*_T_ than deciduous forests ascribed to lower light transmittance of the denser canopy in conifers, in which asymmetric competition for light is dominant [40].

Despite the progress made so far, our quantitative understanding of the allocation patterns of *M*_L_ and *M*_T_ in relation to leaf form and function in trees still remains limited. By analyzing long-term data collected from 1175 tree-dominant large plots located across the Chinese, we address several important questions to the trees growing in Chinese forests: 1) whether there is a general pattern of biomass partitioning for *M*_L_ and *M*_T_ across diverse forestry biomes in terms of location, leaf habit and leaf form; 2) whether there are different allometric scaling exponents between conifer and broadleaved forest; 3) whether the allocation patterns of leaf biomass and total mass vary across evergreen and deciduous forest. Although our contribution here to the ongoing debate about allometry of biomass partitioning is more empirical evidence rather than theories, the answers to the questions will help understand the biomass partitioning pattern in tree-dominant biomes, which is important to future inquiries to mechanisms underlying these scaling exponents appearing to govern important ecological and evolutionary phenomena.

## Materials and Methods

### Data resource and collection

In this research, we used a Chinese forest biomass dataset complied from continuous forest-inventory plots of the Forestry Ministry of China in different time-periods during 1970–1994, published forest reports in over 60 Chinese journals and additional investigations by the author himself [41]. The dataset provides standing biomass and production estimates for all plant components (including stem, branch, leaf, root and total plant mass) associated with climatic characters and age information for the selected 1,266 forest plots covering 17 forest types representative of the entire forest vegetation across China. The database has been applied to abundant researches ascribed to its great contribution to biomass estimation in the past decade. More detailed information describing the dataset can be traced in Luo [41], Ni et al. [42] and Zhang et al. [43]. Here, we described only information relevant to the current analysis.

In the field investigations, “allometric equation method” was widely used. Plant biomass of these forest communities were determined by harvesting quadrates [44]. In the forest plots (0.04–0.5 ha), tree height (*H*, m) and diameter at breast height (*D*, cm) were measured for all trees with *D* greater than 2.5 cm. Six to eight trees of different diameters in a plot were cut down as samples (authorized and approved by Changbai Mountain Forest Ecosystem Research Station, Chinese Academy of Sciences and Huitong Forest Ecosystem Research Station in Changbai Mountain and Guangping, respectively; no endangered or protected species included); the separated stem, branch, leaf and root organs, the combination of whose biomass equaled to plant total mass (*M*_T_), were given a direct measurement. The biomass of the rest trees were estimated by species-specific allometric equations derived from these harvested trees based on *H* and *D* [44], [45]. The equations are always expressed as *M* = a*D* ^b^ or *M* = a(*D* ^2^ *H*)^b^ [41], [43]. Leaf biomass (*M*_L_) estimation was conducted in the growing seasons with fully expanded leaves in early July or August across evergreen and deciduous species [44]. Trees with diameter at breast height (*D*, cm) smaller than 2.5 cm were excluded in the dataset. This method was widely immersed in the database. Moreover, “mean tree method” (chose “mean trees” in plots as samples) and “clear-cutting method” (all trees were cut and measured) were used for biomass estimation as well. Further detailed information about data collection was described in Luo *et al*. [45] and Zhang *et al*. [43].

### Data grouping

#### Group 1

Leaf morphology and physiology are closely associated with environmental variation and species specification. To test whether the *M*_L_ versus *M*_T_ scaling exponents were conserved or varied with these factors, we divided the dataset into five biomes. The biomes were defined according to temperature- and precipitation-based biome classifications in addition to leaf habit and leaf form. Not all observations in the original dataset of Luo [41] had sufficient information to be placed into a biome category. Sufficient data were available only to evaluate relationships for the temperate (including boreal) and subtropical biomes. Therefore, 1175 selected plots of forests in the database were sorted into the following five biomes based on the combination of latitude (temperate/subtropical), leaf habit (evergreen/deciduous) and leaf form (conifer/broadleaf): Biome 1, Temperature Deciduous Coniferous Forest (TDCF), consisting of 48 plots of Temperature *Larix* Forest; Biome 2, Temperature Evergreen Coniferous Forest (TECF), consisting of 168 plots of Boreal/alpine *Picea*-*Abies* Forest, 10 plots of Boreal *Pinus sylvestris* var. *mongolica* Forest, 154 plots of Temperate *Pinus tabulaeformis* Forest; Biome 3, Temperature Deciduous Broadleaved Forest (TDBF), consisting of 165 plots of Temperate Typical Deciduous Broadleaved Forest, 127 plots of Temperate/subtropical Montane *Populus-Betula* Deciduous Forest; Biome 4, Subtropical Evergreen Broadleaved Forest (SEBF), consisting of 238 plots of Subtropical Evergreen Broadleaved Forest (without detailed species information); Biome 5, Subtropical Evergreen Coniferous Forest (SECF), consisting of 66 plots of Subtropical *Pinus massoniana* Forest, 98 plots of Subtropical *Cunninghamia lanceolata* Forest, 46 plots of Subtropical Montane *Pinus yunnanensis* and *P. khasya* Forest, 55 plots of Subtropical montane *Pinus armandii*, *P. taiwanensis* and *P. densada* Forest. Detail information about the biomes, including vegetation, location, climate characters and ages could be traced in Table 1.

**Table 1:** Vegetation, climatic characteristics and ages of biomes in Luo (1996).

Biome	Altitude (m)	Longitude (°)	Latitude (°)	MAT (°C)	MAP (mm)	PET (mm)	Age (years)
TDCF	441–4,240	86.4–131.8	28.6–52.6	−6.2–4.2	370.9–1274.1	340.3–522.9	30–195
TECF	240–4,200	81.1–131.8	26.14–53.0	−6.6–13.9	369.6–1937.3	328.7–820.5	15–350
TDBF	150–3,640	85.2–134.0	25.75–52.5	−5.5–16.0	241.0–1282.8	358.3–939.9	20–222
SEBF	80–4,160	85.2–120.17	20.7–30.25	3.5–24.2	636.6–2323.1	386.4–1131.7	3–200
SECF	10–3,558	85.2–121.57	18.7–36.4	5.7–24.0	369.7–2989.1	503.8–1130.3	15–160
Total	10–4,240	81.1–134.0	18.7–53.0	−6.6–24.2	241.0–2989.1	328.7–1131.7	3–350

*TDCF* Temperate Deciduous Coniferous Forest, *TECF* Temperate Evergreen Coniferous Forest, *TDBF* Temperate Deciduous Broadleaved Forest, *SEBF* Subtropical Evergreen Broadleaved Forest, *SECF* Subtropical Evergreen Cniferous Forest. MAT (°C) stands for Mean Annual Temperature (°C). MAP (mm) stands for Mean Annual Precipitation (mm). PET (mm) stands for Potential Evapotransporation (mm).

#### Group 2

To illustrate the effect of leaf form on biomass partitioning pattern of *M*_L_ and *M*_T_, we divided the dataset (the same as in Group 1) into two categories: conifer and broadleaved forests. Conifer Forest (CF) consisted of 48 plots of Temperature *Larix* Forest, 168 plots of Boreal/alpine *Picea*-*Abies* Forest, 10 plots of Boreal *Pinus sylvestris* var. *mongolica* Forest, 154 plots of Temperate *Pinus tabulaeformis* Forest, 66 plots of Subtropical *Pinus massoniana* Forest, 98 plots of Subtropical *Cunninghamia lanceolata* Forest, 46 plots of Subtropical Montane *Pinus yunnanensis* and *P. khasya* Forest, 55 plots of Subtropical montane *Pinus armandii*, *P. taiwanensis* and *P. densada* Forest. Broadleaved Forest (BF) consisted of 165 plots of Temperate Typical Deciduous Broadleaved Forest, 127 plots of Temperate/subtropical Montane *Populus-Betula* Deciduous Forest, 238 plots of Subtropical Evergreen Broadleaved Forest (without detailed species information).

#### Group 3

To test the influence of leaf habit on the biomass partitioning of *M*_L_ and *M*_T_, we divided the dataset (the same as in Group 1) into two categories: evergreen and deciduous forest. Evergreen Forest (EF) consisted of 168 plots of Boreal/alpine *Picea*-*Abies* Forest, 10 plots of Boreal *Pinus sylvestris* var. *mongolica* Forest, 154 plots of Temperate *Pinus tabulaeformis* Forest, 238 plots of Subtropical Evergreen Broadleaved Forest (without detailed species information), 66 plots of Subtropical *Pinus massoniana* Forest, 98 plots of Subtropical *Cunninghamia lanceolata* Forest, 46 plots of Subtropical Montane *Pinus yunnanensis* and *P. khasya* Forest, 55 plots of Subtropical montane *Pinus armandii*, *P. taiwanensis* and *P. densada* Forest. Deciduous Forest (DF) consisted of 48 plots of Temperature *Larix* Forest, 165 plots of Temperate Typical Deciduous Broadleaved Forest, 127 plots of Temperate/subtropical Montane *Populus-Betula* Deciduous Forest.

### Statistical protocols

As was common in cross-species analysis, data for analyzing the scaling relations of mean individual *M*_L_ and *M*_T_ (Tons/individual), which were obtained as stand mass/density in plots, were log_10_-transformed to normalize the distributions and minimize patterns in residuals [46]. Because functional rather than predictive relationships were sought [47], Model Type II (RMA) regression was used to determine the slope (scaling exponent, *β*) and y-intercept (allometric constant, *K*) of log-log linear functions. A standardized major axis (RMA) fit was the line minimizing sums of squares in X and Y dimensions simultaneously; and these routines were run using the ‘Standardized Major Axis Tests and Routines’ (SMTR) computer package [48]. The software package SMTR was also used to determine whether the numerical values of *β* for log_10_ *M*_L_ vs. *M*_T_ differed between contrasted data subsets. Heterogeneity between SMA slopes within and among biomes was tested via a permutation test in SMATR [48].

## Results

**Figure 1:**
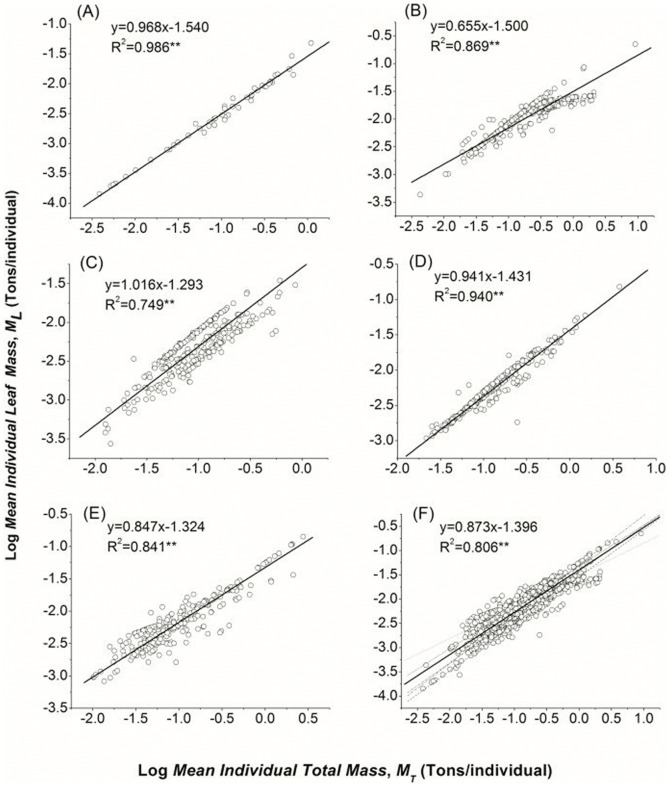
Scaling relations of leaf mass (*M*_L_, Ton/individual) and total biomass (*M*_T_, Ton/individual) in differing forested biomes. *M*_T_ includes leaves, branches, stems and roots. The lines are linear RMA fits to the log-transformed data. For each biome, data include dominant trees from stands of variable age: (A) TDCF, (B) TECF, (C) TDBF, (D) SEBF, (E) SECF. All data are pooled in (F).

For all the forest plots pooled (n = 1175), *M*_L_ increased with *M*_T_ (*P*<0.001, R^2^ = 0.806; Table 2 and Fig. 1) with the scaling exponent of 0.873 (95% CIs: 0.851–0.895), which was statistically higher than a predicted 2/3 or 3/4 and lower than 1.This exponents varied significantly in diverse biomes, with a range of 0.655 to 1.016. TDBF and TDCF had the highest scaling exponents, which were very close to 1. SEBF and SECF had the medium exponents, the values of which were significantly higher than 3/4. TECF shared a value of 0.655, which was close to 2/3 (Table 2; Fig. 1). The average exponents of the five biomes were 0.885, which deviated from 3/4.

**Table 2:** Allometric scaling relationships of leaf mass and total mass across biomes as estimated by standardized Major Axis Estimation and Testing Routines (SMATR).

Biome	*β*	SE	95%CIs	*K*	SE	95%CIs	R^2^	*P*-value	No.
TDCF	0.968^ab^	0.017	0.935, 1.003	−1.540	0.020	−1.581, −1.500	0.986	0.000	48
TECF	0.655^d^	0.013	0.630, 0.681	−1.500	0.012	−1.524, −1.476	0.869	0.000	332
TDBF	1.016^a^	0.030	0.959, 1.077	−1.293	0.033	−1.357, −1.229	0.749	0.000	292
SEBF	0.941^b^	0.015	0.912, 0.972	−1.431	0.015	−1.461, −1.402	0.940	0.000	238
SECF	0.847^c^	0.021	0.807, 0.889	−1.324	0.023	−1.369, −1.278	0.841	0.000	265
Total	0.873	0.011	0.851, 0.895	−1.396	0.012	−1.419, −1.374	0.806	0.000	1175

*β* is the exponent as a consequence of individual *M*_L_ (leaf mass, tons/individual) scales with *M*_T_ (total mass, tons/individual). 95%CIs and SE are confidence intervals and standard error for *β* and *K*, respectively. No. is the number of plots. *TDCF*, *TECF*, *TDBF*, *SEBF*, *SECF* are defined as in Table 1.

**Figure 2:**
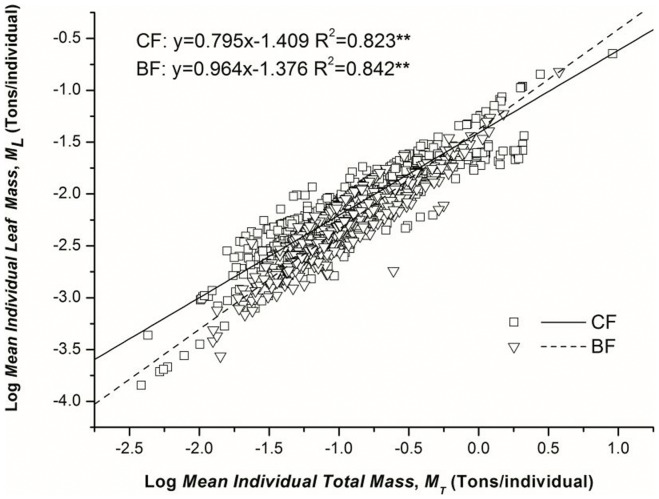
Scaling relations of leaf mass (*M*_L_, Ton/individual) and total biomass (*M*_T_, Ton/individual) in conifer forest (CF) and broadleaved forest (BF). *M*_T_ includes leaves, branches, stems and roots. The lines are linear RMA fits to the log-transformed data.

**Figure 3:**
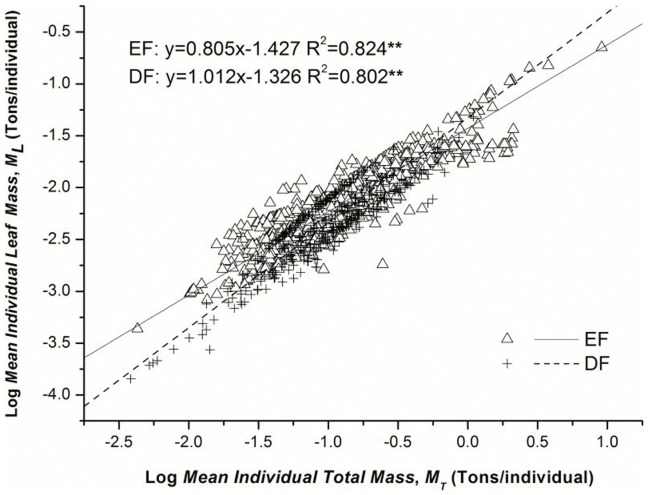
Scaling relations of leaf mass (*M*_L_, Ton/individual) and total biomass (*M*_T_, Ton/individual) in evergreen forest (EF) and deciduous forest (DF). *M*_T_ includes leaves, branches, stems and roots. The lines are linear RMA fits to the log-transformed data.

Regressions performed in Group 2 showed that the biomass allocation pattern of *M*_L_ and *M*_T_ varied from conifer to broadleaved forests. The exponent of CF (0.795^b^) was lower than BF (0.964^a^). (Table 3; Fig. 2)These results were mostly consistent with the findings in Group 1 that in both temperate and subtropical forests, the scaling exponents of broadleaved biomes (TDBF, 1.016^a^; SEBF, 0.941^b^) were commonly higher than the conifer ones (SECF, 0.847^c^, TECF, 0.655^d^; except TDCF, 0.968^ab^) (Table 2; Fig. 1). Regressions assessed in Group 3 suggested that the scaling exponents of leaf mass and total mass of deciduous forests (1.012^b^) were higher than evergreen forests (0.805^a^) (Table 4; Fig. 3). These findings agreed with the observation that the scaling exponents in deciduous forests (TDBF, 1.016; TDCF, 0.968) were higher than in evergreen forests (SEBF, 0.941; SECF, 0.847; TECF, 0.655) (Table 2; Fig. 1).

**Table 3:** Allometric scaling relationships of leaf mass and total mass between conifer and broadleaved forests as estimated by standardized Major Axis Estimation and Testing Routines (SMATR).

Category	*β*	SE	95%CIs	*K*	SE	95%CIs	R^2^	*P*-value	No.
CF	0.795^b^	0.018	0.770, 0.821	−1.409	0.014	−1.436, −1.383	0.823	0.000	645
BF	0.964^a^	0.015	0.932, 0.998	−1.376	0.017	−1.410, −1.341	0.842	0.000	530

*β* is the exponent as a consequence of individual *M*_L_ (leaf mass, tons/individual) scales with *M*_T_ (total mass, tons/individual). 95%CIs and SE are confidence intervals and standard error for *β* and *K*, respectively. No. is the number of plots. *CF* and *BF* represent Conifer Forest and Broadleaved Forest, respectively.

**Table 4:** Allometric scaling relationships of leaf mass and total mass between evergreen and deciduous forests as estimated by standardized Major Axis Estimation and Testing Routines (SMATR).

Category	*β*	SE	95%CIs	*K*	SE	95%CIs	R^2^	*P*-value	No.
EF	0.805^b^	0.012	0.782, 0.828	−1.427	0.012	−1.450, −1.404	0.824	0.000	835
DF	1.012^a^	0.023	0.965, 1.061	−1.326	0.027	−1.379, −1.272	0.802	0.000	340

*β* is the exponent as a consequence of individual *M*_L_ (leaf mass, tons/individual) scales with *M*_T_ (total mass, tons/individual). 95%CIs and SE are confidence intervals and standard error for *β* and *K*, respectively. No. is the number of plots. *EF* and *DF* represent Evergreen Forest and Deciduous Forest, respectively.

## Discussion

Scaling has been treated as a particularly powerful tool in physical sciences for revealing “emergent” phenomena in complex systems [49]. WBE theory aims to provide the broadest coverage of allometric phenomena with 1/4 rules. West *et al*. [9], [10] and Enquist and Niklas [12] and Niklas [4] predicted that the leaf mass scaled as the 3/4 power of body mass based on an internalized fractal delivery networks for energy and mass transfer. Although their predictions were validated with a global database [11], [14], it was not the same case in our results. Our results showed that the biomass partitioning of leaf organs and total plants were much variable across diverse forested biomes. The discrepancy may partly result from the different scale-levels of investigations. The data cited by Enquist [14] spans over 20 orders of magnitude of body size from unicellular to multi-cellular plants. The large-scale process tends to show relative independence of phylogenetic affiliation, growth habit and abiotic environmental features [50]. In contrast, the tight clustering of our data covers only three orders of magnitude in standing tree mass [41], which necessarily accentuate the biological or environmental characters in local sample [51]. Despite the potential statistical weakness in local-sample regression [11], the limitation is of minor relevance to our result, as all the regressions in our report have high r^2^ values (Table 2; Fig. 1) [31], [32], [34], [52]. However, the distribution of the exponents in our result (from 0.655 to 1.016) was similar to the combining 95% CIs of scaling exponents regressed for angiosperms and gymnosperms from the global database (from 0.646 to 0.946) [14]. These ranges were consistent with the intra-specific scaling relationships predicted by Kozlowski *et al*. [53], which was from 2/3 to 1, addressing the impact of species specification on the biomass partitioning pattern. In fact, WBE has been modified with the consideration of ontogeny and taxonoxy [54], [55]. Price et al.[15], [55], [56] suggested a signigicant variability between speceis and predicted that the the scaling exponent should fall along a continuum of variation based on more extension models of WBE, such as leaf venation networks. All these attempts have greatly promoted WBE theories to embrace more varibable emperical findings.

As sessile organisms, plants have to be evolved with highly variable leaf form and function as both cause and consequence for adaptive strategy in response to species variety and environmental heterogeneity. In our research, the differing leaf mass and total mass partitioning relationships within groups indicated that leaf form and leaf habit played essential roles in adjusting the biomass allocation patterns (Table 2, 3, 4; Fig. 1, 2, 3). The conifer forests always produced needle-like leaves with lower special leaf area (SLA). SLA is positively correlated with potential photosynthetic rate, decomposition rate, leaf toughness and relative leaf growth rate [57]. Broadleaves with higher SLA can provide high metabolism (gas change rates) and energy capture (light harvesting and CO_2_ fixation) per unit leaf mass [39], [46]. The discrepancy of SLA is consistent with the different resource consumption strategies adapted by CF and BF, which are resource-conserving and resource-demanding, respectively [58]. That may explain why the scaling exponent of leaf mass and total mass was lower in CF than in BF (Table 3; Fig. 2). In addition, the conifer trees commonly achieve their growing space by more lateral and less vertical oriented expansion, leading to a broom-like crown. In contrast, the broadleaved trees always arrange their leaf area in an umbrella-like shape, which demands more space compared to broom-like crowns [59]. The different crown structure challenges the assumption by WBE that leaf area ∝ leaf mass, which may as well cause a failure of universal biomass partitioning. We also observed that the *M*_L_ versus *M*_T_ scaling exponents were commonly lower in evergreen forest biomes than those in deciduous ones (Table 2, 4; Fig. 1, 3). These phenomena are consistent with the adaptive strategies of evergreen and deciduous functional types in warm temperate to tropical regions. Evergreen trees always produce leaves that have a longer lifespan, lower SLA and higher tensile strength than deciduous trees. A longer lifespan of leaves requires investment in structural integrity and/or defense against disturbances, especially with resource constraint, such as sun light [60]. Thereby the evergreen plant promotes greater allocation of biomass to structural rather than metabolic components [61], resulting in lower scaling exponents of *M*_L_ and *M*_T_ as plant grows. These phenomena explicate the patterns of leaf mass and total mass partitioning occurring in deciduous and evergreen forests. Noticeably, the scaling exponents of leaf mass and total mass are higher deciduous forests than in evergreen forests, in spite of conifer or broadleaved species (Table 2; fig. 1), suggesting the importance of leaf lifespan in determining biomass allocation patterns. Although the forests we selected commonly distribute in temperature or subtropical area, the variation of allocation patterns keeps consistent with forests in tropical dry forests [39]. Therefore, the influence of leaf form and functions could not be neglected in metabolism and the construction of biomass allocation patterns.

Forest is the most important carbon pool in terrestrial ecosystem. Given the common perception of leaves as engines of metabolic activity, scaling relation of *M*_L_ with *M*_T_ performs as a consequence of the synergy between physiological, biophysical and evolutionary constraints on leaf phenotypes of all species in terrestrial ecosystems. In the present research, the majority of regression curves failed the test for slope homogeneity, suggesting there is hardly a uniform pattern of biomass partitioning for the differing forests in terms of leaf form and function. Although the numerical values do not conform to a WBE prediction, a simple right or wrong is not our intention. Our analysis in this research mainly focused on the variations within the sorted forests, while in-depth understanding of mechanism underlying the allometric biomass patterns, especially the relations with leaf morphological, physiological and anatomical traits, will be explored in future work.

## Conclusions

Biomass partitioning is considered a strong driver of the capacity of plants to take up carbon, water and nutrients for future use. A visual and statistical examination of our collective data from a Chinese forest biomass dataset empirically reveals a striking heterogeneity in scaling laws of *M*_L_ with *M*_T_ across diverse tree-dominated biomes, between conifer and broadleaved forests, and between evergreen and deciduous forests. The results of the present study highlight the effects of leaf form and function on the behavior of individuals and consequently on populations and communities, questioning the application of unique scaling law of biomass partitioning to all terrestrial plants. Many details of plant morphology, physiology as well as ecology characters (just like light, water and nutrient availability, plant density) can be incorporated into the allometric coefficients to link pattern and process across multiple scales and biological organization. This work may invoke further research about the morphological, physiological, biochemical and phylogenic mechanism underlying scaling.
